# ZDHHC5 Mediates Immune Dysregulation Driving Generalized Anxiety Disorder Risk

**DOI:** 10.1002/brb3.70818

**Published:** 2025-09-02

**Authors:** Fuqiang Sun, Gengchen Lu, Nannan Li, Kun Dai, Zhe Ren, Ruimin Ma, Yifei Feng, Shuo Zong, Bin Cheng

**Affiliations:** ^1^ College of Acupuncture and Tuina Shandong University of Traditional Chinese Medicine Jinan Shandong China; ^2^ Independent Researcher Xiamen Fujian China; ^3^ Department of Acupuncture Affiliated Hospital of Shandong University of Traditional Chinese Medicine Jinan Shandong China

**Keywords:** generalized anxiety disorder (GAD), immune cells, Mendelian randomization (MR), palmitoylation

## Abstract

**Objective:**

To investigate genes associated with palmitoylation modification implicated in generalized anxiety disorder (GAD), and to elucidate the mechanistic roles of these genes via immune cell‐mediated pathways.

**Methods:**

Utilizing large‐scale genetic datasets, genes categorized as palmitoylation‐expression quantitative trait loci (eQTL) were identified by intersecting palmitoylation‐related genes (as compiled from the most recent reviews) with genome‐wide eQTL datasets. A two‐sample Mendelian randomization (MR) methodology was applied to investigate the causal relationship between palmitoylation‐eQTL genes and GAD, with further verification conducted through summary‐data‐based Mendelian randomization (SMR) to establish the final target genes. Subsequently, the underlying mechanisms of these genes were examined through immune cell‐mediated processes.

**Results:**

Twenty‐two palmitoylation‐eQTL gene loci were identified, among which five demonstrated a notable link to GAD: PPT2 (odds ratio [OR] = 0.844, *p* = 0.007), ZDHHC5 (OR = 1.101, *p* = 0.017), ZDHHC13 (OR = 1.175, *p* < 0.001), ZDHHC20 (OR = 0.874, *p* = 0.003), and ZDHHC24 (OR = 0.806, *p* < 0.001). ZDHHC5 and ZDHHC13 were validated as final target genes through SMR analysis. Mediation MR analysis revealed that SSC‐A on CD4+ T cells (mediation proportion: 36.4%) served as a mediating factor for ZDHHC5.

**Conclusion:**

The findings indicate that ZDHHC5 contributes to the pathogenesis of GAD by modulating SSC‐A on CD4+ T cells, thereby offering novel targets for the investigation of GAD pathophysiology.

## Graphical abstract

Mendelian randomization identifies palmitoylation genes driving generalized anxiety disorder (GAD) risk, notably ZDHHC5 and ZDHHC13. ZDHHC5 promotes GAD pathogenesis by modulating SSC‐A on CD4+ T cells, revealing novel immune‐mediated mechanisms and therapeutic targets for GAD.

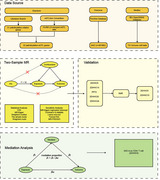

## 1 Introduction

Generalized anxiety disorder (GAD) represents a prevalent anxiety condition, defined by persistent and excessive worry concerning daily life, often accompanied by somatic symptoms including sleep disturbances, restlessness, muscle tension, gastrointestinal discomfort, and chronic headaches (Locke et al. 2015, Association AP 2013). Individuals diagnosed with GAD are at an elevated risk of suicide and display increased susceptibility to cardiovascular complications and mortality (DeMartini et al. 2019). Epidemiological data reveal that the lifetime prevalence of GAD among Americans aged 13 years and older is 4.3% (Kessler et al. 2012). In the current multifaceted social context, particularly following the COVID‐19 pandemic, the intensification of stress across domains such as personal life, education, and employment has contributed to a further escalation in GAD prevalence (Collaborators 2021). Currently, the primary therapeutic approaches for GAD consist of pharmacological treatment and psychotherapy. Selective serotonin reuptake inhibitors (SSRIs) and serotonin‐norepinephrine reuptake inhibitors (SNRIs) represent the first‐line pharmacotherapy for generalized anxiety disorder (GAD) (Penninx et al. 2021), demonstrating significant improvement in anxiety symptoms compared to placebo (Kopcalic et al. 2025). However, treatment is limited by adverse effects leading to discontinuation and a delayed onset of action (2‐4 weeks) (Penninx et al. 2021, Kopcalic et al. 2025). While benzodiazepines exhibit rapid efficacy, they are associated with relapse upon discontinuation and a significant risk of dependence (Penninx et al. 2021). Psychotherapeutic interventions have been shown to enhance functional outcomes and quality of life, with evidence supporting sustained long‐term effectiveness (Mirchandaney et al. 2022, Papola et al. 2024). Nevertheless, psychotherapy is often time‐intensive and constrained by a limited availability of qualified practitioners. GAD imposes a considerable burden in terms of disability, psychological distress, and societal economic costs (Hendriks et al. 2016). Consequently, understanding the biological mechanisms responsible for GAD remains essential in identifying novel treatment approaches.

The development of GAD has also been associated with dysregulation of immune system function. Laboratory evidence suggests that aberrant secretion of inflammatory markers—including tumor necrosis factor‐alpha (TNF‐α), interleukin‐6 (IL‐6), and interferon‐gamma (IFN‐γ)—potentially drives heightened anxiety levels (Ogłodek et al. 2015, Tang et al. 2018). Notably, antidepressants demonstrate anti‐neuroinflammatory properties—SSRIs like fluvoxamine reverse inflammatory damage through inhibition of high‐mobility group box 1 (HMGB‐1) and activation of sirtuin 1/glutathione peroxidase 4 (SIRT‐1/GPX‐4) (Tepebaşı et al. 2025), whereas SNRIs, including levomilnacipran, ameliorate synaptic dysfunction via brain‐derived neurotrophic factor/tropomyosin receptor kinase B–phosphoinositide 3‐kinase/protein kinase B/mammalian target of rapamycin (BDNF/TrkB–PI3K/Akt/mTOR) signaling while suppressing cytokine production (Wu et al. 2024).

Palmitoylation, typically referring to S‐palmitoylation—also known as S‐acylation—constitutes a highly prevalent post‐translational protein modification, potentially influencing 10%–20% of the human proteome (F et al. 2024). This lipid modification has been shown to modulate protein subcellular localization, membrane association, structural stability, and interactions with other proteins, thereby affecting various cellular physiological processes (Jiang et al. 2018). Aberrant palmitoylation has emerged as a significant element in the progression of various pathological conditions, encompassing neurological disorders, inflammatory conditions, infections, and malignancies (Zhou et al. 2023, Jin et al. 2021). Moreover, abnormalities in protein palmitoylation are particularly implicated in brain‐related disorders, with 9 out of the 24 ZDHHC enzymes reportedly associated with neurological diseases (Zaręba‐Kozioł et al. 2018). Notably, the reversible nature of palmitoylation allows for precise temporal and spatial regulation of protein trafficking at membrane interfaces (F et al. 2024), thereby rendering it an attractive therapeutic candidate for multiple disease states. Critically, palmitoylation functions as an essential modulator in immune signal transduction cascades (Lu et al. 2019, Mukai et al. 2016). On the basis of these findings, a causal association between palmitoylation and GAD has been postulated, with immune cells proposed to mediate the interaction between these two factors.

Mendelian randomization (MR) functions as an analytical method in genetic epidemiology that utilizes genetic variations to establish causative associations between exposures and outcomes, facilitating the estimation of direct effects, indirect effects, and mediation proportions (Emdin et al. 2017, Carter et al. 2021). In contrast to conventional non‐instrumental variable (IV) mediation methods, MR analysis minimizes biases resulting from confounding and measurement errors among exposures, mediators, and outcomes (Carter et al. 2021). By integrating genome‐wide association study (GWAS) data with expression quantitative trait loci (eQTL), target genes linked to risk variants can be identified through causal inference methodologies (Kreitmaier et al. 2023). In this study, a two‐sample MR framework was implemented, employing genetic IVs robustly associated with palmitoylation‐related variants identified via GWAS and cis‐eQTL analyses. Additionally, a two‐step MR approach was applied to quantify the potential mediating roles of immune cell traits within the hypothesized causal pathway between palmitoylation processes and GAD. This analytic strategy may offer new perspectives on the pathogenic mechanisms underlying GAD.

## 2 Materials and Methods

### 2.1 Research Design (The Workflow of the Present Study is Depicted in Figure 1)

First, by retrieving recent palmitoylation reviews, extract genes explicitly related to palmitoylation. Second, candidate palmitoylation gene targets for GAD were identified using MR methodologies applied to 15,695 locally pruned eQTL data entries, encompassing genes encoding drug targets or proteins associated with drug targets. Subsequently, summary‐data‐based Mendelian randomization (SMR) was conducted to validate the identified palmitoylation gene targets. Finally, the potential mediating effects of 731 immune cell traits were assessed through a two‐step MR mediation analysis.

### 2.2 Data Sources

#### 2.2.1 eQTL

Blood cis‐eQTL data were procured from the eQTLGen Consortium, specifically from the dataset available at https://molgenis26.gcc.rug.nl/downloads/eqtlgen/cis‐eqtl/2019‐12‐11‐cis‐eQTLsFDR‐ProbeLevel‐CohortInfoRemoved‐BonferroniAdded.txt.gz. Local clumping was applied to all raw data to eliminate linkage disequilibrium (LD) using PLINK v1.90 (*p*‐value < 5.0 × 10^−8^, clumping window: 10,000 kb, r^2^ < 0.1), yielding 15,695 usable eQTL entries.

Disease: GWAS summary statistics for GAD were procured from the FinnGen database (https://storage.googleapis.com/finngen‐public‐data‐r12/summary_stats/release/finngen_R12_F5_GAD.gz), corresponding to disease ID F5_GAD. Covariate adjustment included sex, age, the first 10 principal components, Finngen chip version (1 or 2), and genotyping batch effects (Kurki et al. 2023). The dataset comprised 7,148 GAD cases and 444,414 controls. For MR analysis, we extracted effect sizes, standard errors, effect alleles, non‐effect alleles, allele frequencies, and P‐values for all SNPs.

#### 2.2.2 SMR Data

Analytical tools and datasets were procured from the publicly available SMR software portal (https://yanglab.westlake.edu.cn/software/smr/#DataResource), along with cis‐eQTL data derived from the eQTLGen Consortium (https://www.eqtlgen.org/cis‐eqtls.html).

#### 2.2.3 Immune Cell Traits

Data on 731 immune cell traits (Ebi‐a‐GCST0001391 to Ebi‐a‐GCST0002121) was procured from the IEU OpenGWAS project database (https://gwas.mrcieu.ac.uk/). These phenotypes spanned seven major cell types: B cells, dendritic cells, mature T cells, monocytes, myeloid cells, TBNK, and regulatory T cells. The data encompassed absolute and relative cell counts, median fluorescence intensity indicating surface antigen expression, and morphological characteristics (Orrù et al. 2020). All samples were derived from Western populations. For each phenotype, we downloaded the full GWAS summary statistics file containing effect sizes, standard errors, alleles, allele frequencies, and *P*‐values.

This investigation represents a secondary analysis of open‐access datasets, all of which are publicly available. Consequently, further ethical clearance was not deemed necessary.

### 2.3 MR Design

In this study, genome‐wide eQTL data were subjected to local pruning based on LD, after which MR analyses were executed using the TwoSampleMR R package to explore the causal associations between palmitoylation‐eQTL genes and GAD, 731 immune cell traits and GAD, as well as target palmitoylation‐eQTL genes and immune cell traits that are causally associated with GAD. The SMR method was employed to assess whether gene expression levels mediated the causal relationships between single nucleotide polymorphisms (SNPs) and GAD (Zhu et al. 2016).

### 2.4 Selection of Genetic IVs

MR utilizes genetic variants as proxies for risk factors. The genetic IVs incorporated into the analysis were required to fulfill three fundamental assumptions (Davies et al. 2018): (1) the IV must demonstrate a significant association with the exposure factor; (2) the IV must not be associated with any confounders related to the outcome; and (3) the IV must influence the outcome exclusively by modulating the exposure factor, rather than via alternative pathways. SNPs deemed meaningful were selected as IVs according to the following criteria: (1) a stringent significance threshold was applied, allowing only SNPs with *P*‐values below the genome‐wide significance level (5.0 × 10 − 8) to be retained (Yuan et al. 2023); (2) local LD pruning was carried out utilizing a threshold of r^2^ < 0.1 and a clustering window of 10,000 kb (Gkatzionis et al. 2023); (3) SNPs with incompatible alleles between exposure and outcome datasets (e.g., A/G vs. A/C) were discarded, and allele frequencies were applied to determine the positive strand for palindromic SNPs, or such SNPs were excluded outright if allele frequency data were unavailable; (4) F‐statistics were computed to assess the strength of the IVs in the MR framework, with F > 10 indicating a low likelihood of weak instrument bias (Burgess et al. 2017). Consequently, IVs with F < 10 were eliminated. (5) To mitigate reverse causation bias, we applied Steiger filtering within the TwoSampleMR package. This tests the directionality of causal effects by comparing the proportion of variance explained (R^2^) for each SNP in exposure‐outcome pairs. Only SNPs showing a significantly stronger association with the exposure than the outcome (*p* < 0.05) were retained for the final analysis.

### 2.5 Statistical Analysis

In this MR study, the inverse‐variance weighted (IVW) approach was predominantly applied, as it is widely regarded as the deterministic standard for assessing causal relationships and is considered the most reliable approach for evaluating the presence of causal effects (Burgess et al. 2019). When statistically significant differences were observed via the IVW method (*p* < 0.05), the corresponding results were regarded as potential indicators of causal relationships with exposure factors. To generate robust causal estimates under varying assumptions, several complementary approaches were additionally employed, including MR‐Egger, weighted median, simple mode, and weighted mode. The causal association between palmitoylation and GAD was assessed utilizing odds ratios (OR) alongside their associated 95% confidence intervals (CI).

### 2.6 Sensitivity Analysis

Sensitivity analysis encompassed both horizontal pleiotropy and heterogeneity testing. The intercept of the MR‐Egger regression model was utilized to evaluate horizontal pleiotropy. A non‐significant intercept (*p* > 0.05) was interpreted as evidence for the absence of substantial horizontal pleiotropy in the MR framework (Hemani et al. 2018, Zou et al. 2021). Analysis of SNP heterogeneity utilized Cochrane's Q test through the MR‐Egger model (Bowden et al. 2018). Upon detection of significant heterogeneity (*p* < 0.05), adjustments were made through implementation of a random‐effects IVW model. In addition, leave‐one‐out analysis was performed by systematically excluding each SNP locus in turn and re‐executing the MR analysis to assess the contribution of individual SNPs to the overall outcome, thus assessing result robustness. The MR‐PRESSO methodology was applied to ascertain outliers and determine their impact on the analytical outcomes. The significant global pleiotropy was detected (Global Test *p* < 0.05), the analysis was rerun after removing the identified outlier SNPs, and the corrected causal estimate was reported alongside the original.

### 2.7 SMR Validation

The association between genes and phenotypes was assessed using SMR analysis with the SMR software (version 1.3.1), in which PSMR values and effect sizes were calculated. Potential pleiotropic interferences were eliminated through HEIDI testing (where *P* ≥ 0.05 indicated no significant evidence of pleiotropy), and significant genes exhibiting consistent directional effects were validated via eQTL analysis. Targets lacking statistical significance in SMR (*P*_SMR_ ≥ 0.05) or showing evidence of pleiotropy (*P*_HEIDI_ < 0.05) were subsequently excluded from further mediation analysis.

### 2.8 Mediation MR Analysis

In the preliminary MR analysis, the total causal effect (*β*_all_) of each validated palmitoylation‐eQTL gene target on GAD was estimated. Within this analytical framework, immune cell traits were initially treated as exposures to identify those exhibiting significant causal associations (*p* < 0.05) with GAD, and their causal effects (*β*_2_, representing the independent influence of immune cell traits on GAD) were subsequently estimated. Thereafter, a two‐sample MR analysis was executed employing target palmitoylation‐eQTL genes as exposures and the previously identified immune cell traits as outcomes, quantifying the corresponding effect sizes (β_1_, indicating the regulatory influence of palmitoylation‐eQTL genes on immune cell traits). Finally, mediation effects were calculated using effect estimates derived exclusively from IVW analyses. The product of coefficients method was applied to compute the mediation effect (*β*_1_ × *β*_2_) and its proportion relative to the total effect (*β*_1_ × *β*_2_ / *β*_all_), with a predefined threshold of ≥ 20% for mediation proportion adopted as the criterion for statistical significance.

### 2.9 Analysis Software and Technical Support

All data processing was conducted in RStudio (version 4.4.2). A variety of R packages, including “Mendelian Randomization,” “MRPRESSO,” “TwoSampleMR,” “ggplot2,” “foreach,” “data.table,” and “multiple.smr” were employed to perform the statistical analyses. PLINK software (version v1.90) was utilized to implement local LD pruning. Statistical significance was determined at p < 0.05. SMR software version 1.3.1 (https://cnsgenomics.com/software/smr/#Overview) was adopted for allele alignment and subsequent analysis, wherein default system commands were executed to verify SMR analyses.

## 3 Results

### 3.1 Palmitoylation‐eQTL Genes

Through a retrospective examination of the literature concerning palmitoylation, 31 validated palmitoylation‐related genes were identified (Chen et al. 2024, Li et al. 2023, Chamberlain and Shipston 2015). These genes were subsequently intersected with 15,695 eQTL data points derived following local LD pruning, which led to the identification of 22 co‐localized genes (Figure 2): PPT1, PPT2, ZDHHC1, ZDHHC2, ZDHHC3, ZDHHC4, ZDHHC5, ZDHHC6, ZDHHC7, ZDHHC8, ZDHHC11, ZDHHC12, ZDHHC13, ZDHHC14, ZDHHC16, ZDHHC17, ZDHHC18, ZDHHC19, ZDHHC20, ZDHHC21, ZDHHC23, and ZDHHC24.

**FIGURE 1:**
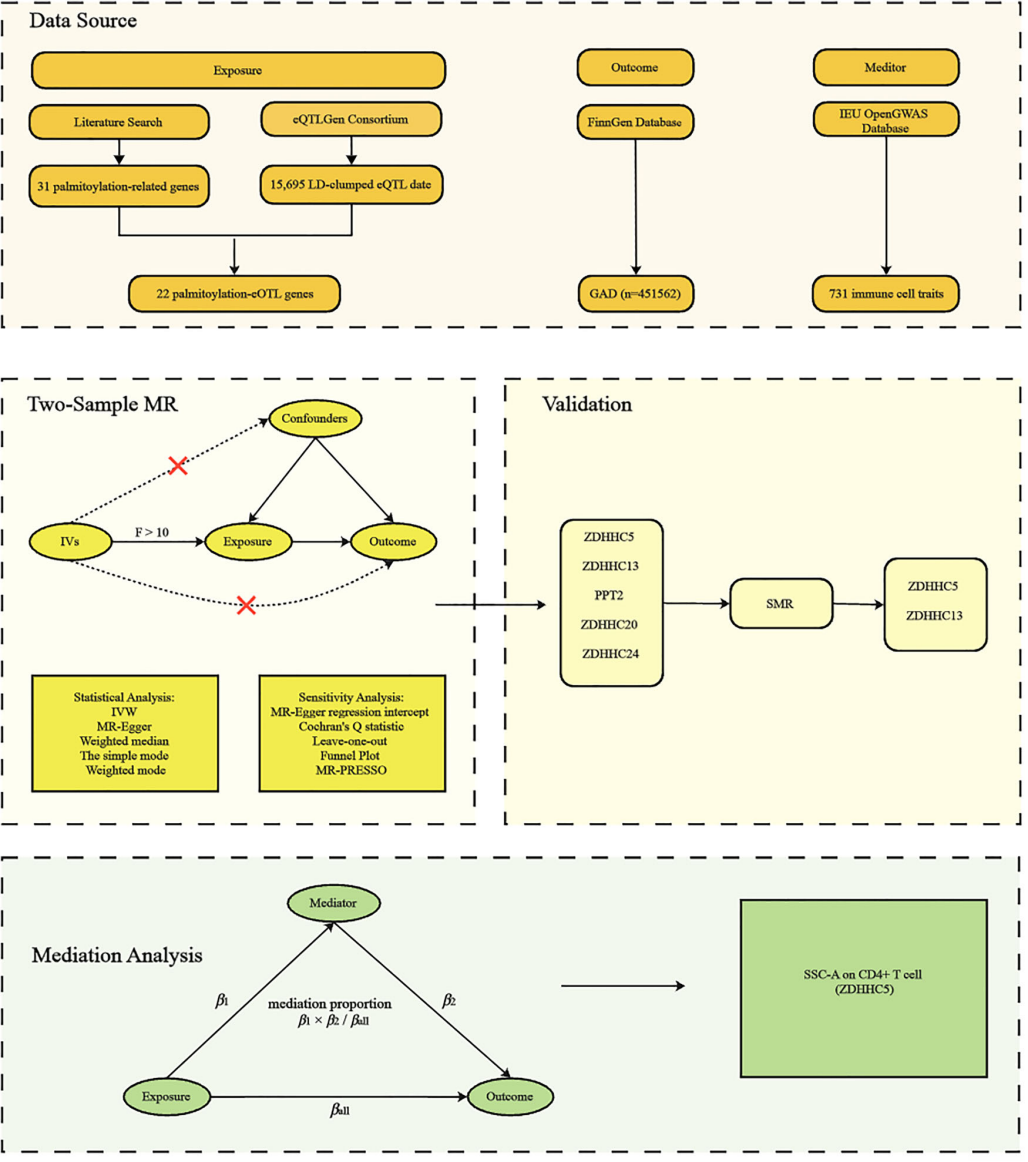
Flowchart of the study process.

**FIGURE 2:**
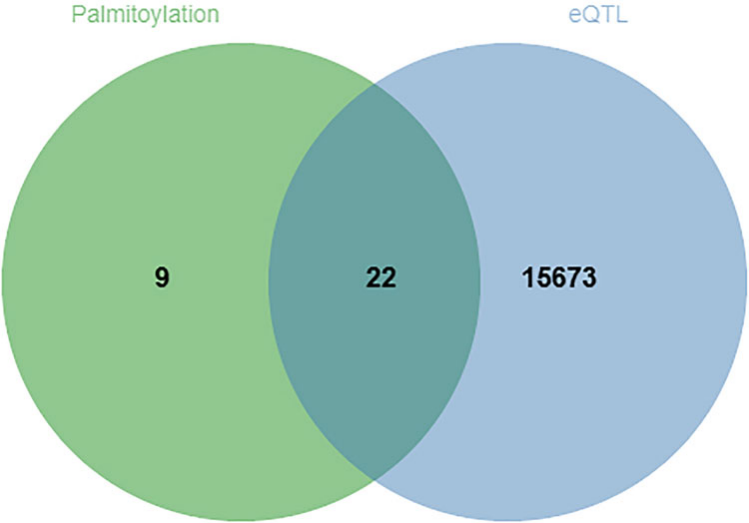
Venn diagram of eQTL and palmitoylation‐related genes.

### 3.2 IV Selection

Based on the predefined screening criteria, 5 palmitoylation‐eQTL genes exhibiting strong associations with GAD were identified from the pool of 22 candidate genes: PPT2, ZDHHC5, ZDHHC13, ZDHHC20, and ZDHHC24. Specifically, 5 SNPs were found to be strongly associated with PPT2, 26 with ZDHHC5, 20 with ZDHHC13, 19 with ZDHHC20, and 14 with ZDHHC24 (Supplementary Table S1).

### 3.3 Effect of Palmitoylation on GAD

**FIGURE 3:**
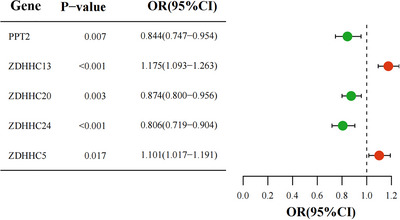
Forest plot of causal effect estimates for palmitoylation‐eQTL genes on GAD using IVW method.

The specific effects of each SNP on GAD are presented in Supplementary Table S2. The IVW analysis indicated bidirectional effects of palmitoylation‐eQTL genes on GAD risk: elevated expression levels of ZDHHC5 and ZDHHC13 were linked to an increased risk (ZDHHC5 OR = 1.101, 95% CI: 1.02 − 1.18, *p* = 0.017; ZDHHC13 OR = 1.175, 95% CI: 1.093 − 1.263, *p* < 0.001), whereas increased expression of PPT2, ZDHHC20, and ZDHHC24 was found to confer protective effects (PPT2 OR = 0.844, 95% CI: 0.747 − 0.954, *p* = 0.007; ZDHHC20 OR = 0.874, 95% CI: 0.800 − 0.956, *p* = 0.003; ZDHHC24 OR = 0.806, 95% CI: 0.719 − 0.904, *p* < 0.001) (Supplementary Table S3) (Figure 3).

The results of Cochran's Q test indicated no significant heterogeneity between the following genes and GAD: PPT2 (Q = 3.580, *p* = 0.311), ZDHHC5 (Q = 23.478, *p* = 0.492), ZDHHC13 (Q = 15.687, *p* = 0.614), ZDHHC20 (Q = 6.613, *p* = 0.988), and ZDHHC24 (Q = 6.217, *p* = 0.905). Moreover, the intercept term analysis from MR‐Egger regression demonstrated no indication of horizontal pleiotropy effects (Table 1). The MR‐PRESSO analysis identified no significant outliers. Hence, no additional correction was deemed necessary (Table 1). Visual inspection of the funnel plots revealed no asymmetry in the distribution of SNP‐specific effect estimates across all genes (Supplementary Figure S1c–S5c). The leave‐one‐out analysis suggested that sequential exclusion of individual SNPs yielded IVW results that remained largely consistent with those derived from the full SNP set (Supplementary Figure S1d–S5d).

**TABLE 1:** MR analysis of palmitoylation‐eQTL genes for GAD: tests for horizontal pleiotropy and heterogeneity.

Gene	Outcome	Cochran's Q test		MR‐Egger regression		MR‐PRESSO analysis	
		Cochran's Q statistic	*p*‐value	MR‐Egger intercept	*p*‐value	MR‐PRESSO RSSobs	*p‐*value
PPT2	GAD	3.580	0.311	−0.035	0.609	5.398	0.504
ZDHHC5	GAD	23.478	0.492	−0.010	0.392	27.526	0.480
ZDHHC13	GAD	15.687	0.614	−0.003	0.838	16.498	0.746
ZDHHC20	GAD	6.613	0.988	0.005	0.712	7.633	0.991
ZDHHC24	GAD	6.217	0.905	−0.015	0.306	8.400	0.899

### 3.4 SMR Validation

Through SMR verification, statistically significant differences were identified in ZDHHC5 (*P*_SMR_ = 0.008) and ZDHHC13 (*P*_SMR_ = 0.003), with no significant heterogeneity detected by the HEIDI test (*P*_HEIDI_ > 0.05), indicating robust findings (Table 2). The OR for ZDHHC5 was 1.205 (95% CI: 1.050 − 1.384), and that for ZDHHC13 was 1.193 (95% CI: 1.064 − 1.338), implying that both may contribute to increased GAD risk. The significant SMR findings, which were aligned with the directionality of the prior MR analysis, further support their potential causal involvement in GAD. Additionally, eQTL analysis revealed that the expression of both genes was markedly regulated (*P*_eQTL_ < 0.001), further reinforcing their association with GAD. In contrast, no statistical significance was observed for PPT2, ZDHHC20, or ZDHHC24 (*P*_SMR_ > 0.05). Consequently, ZDHHC5 and ZDHHC13 appear to be more reliable candidate genes meriting further in‐depth investigation.

**TABLE 2:** SMR validation results of palmitoylation‐eQTL genes associated with GAD.

Gene	CHR	Beta_SMR_	SE_SMR_	*P*_SMR_	*P*_HEIDI_	SNPs_HEIDI_	*P*_eQTL_	*P*_GWAS_	OR (95% CI)
PPT2	6	− 0.198	0.103	0.056	0.435	20	< 0.001	0.052	0.821 (0.670 − 1.005)
ZDHHC13	11	0.177	0.059	0.003	0.555	20	< 0.001	0.002	1.193 (1.064 − 1.338)
ZDHHC5	11	0.187	0.071	0.008	0.115	20	< 0.001	0.008	1.205 (1.050 − 1.384)
ZDHHC24	11	− 0.107	0.144	0.457	0.570	20	< 0.001	0.457	0.898 (0.677 − 1.192)
ZDHHC20	13	− 0.108	0.077	0.160	0.365	20	< 0.001	0.159	0.898 (0.772 − 1.043)

Abbreviations: BetaSMR = estimated effect size in the SMR analysis, representing the standardized effect of a one‐unit increase in gene expression on GAD risk; CHR = chromosome number where the gene is located; *P*eQTL = *p*‐value from the eQTL analysis, reflecting the association strength between gene expression and SNPs; *P*GWAS = *p*‐value from GWAS for the corresponding SNP, indicating its association significance with GAD; PHEIDI = *p*‐value from the HEIDI test, evaluating heterogeneity of instrumental variables (*p* > 0.05 indicates no heterogeneity, supporting the robustness of causal inference); PSMR = *p*‐value for significance in the SMR analysis (values marked as “< 0.001” if *p* < 0.001); SESMR = standard error of the effect size; SNPsHEIDI = number of SNPs used in the HEIDI test.

### 3.5 Mediation Analysis

#### 3.5.1 Effects of Immune Cells on GAD

Screening of immune cells associated with GAD: A two‐sample MR methodology was implemented with 731 immune cell traits functioning as exposures and GAD serving as the outcome. Overall, 36 immune cell traits demonstrated a notable link to GAD (Supplementary Table S4).

#### 3.5.2 Effects of Target Genes ZDHHC5 and ZDHHC13 on Immune Cells

Screening of immune cell traits related to target genes ZDHHC5 and ZDHHC13: batch two‐sample MR analyses were conducted by designating ZDHHC5 and ZDHHC13 as exposures and the 36 immune cell traits obtained from the previous step as outcomes. Consequently, 7 immune cell traits associated with ZDHHC5 (Supplementary Table S5) and 12 associated with ZDHHC13 (Supplementary Table S6) were identified.

#### 3.5.3 Effects of Target Genes ZDHHC5 and ZDHHC13 on GAD through Immune Cells

Based on the screening results derived from the data presented in Tables 3 and 4, mediation effects with directional inconsistencies relative to total effects were excluded, and results exhibiting mediation effect proportions of no less than 20% (*β*_12_p ≥ 20%) were retained. In instances where multiple mediation effects satisfied this threshold, the effect with the greatest mediation proportion was prioritized. The trait that met these criteria was SSC‐A on CD4^+^. According to the GWAS catalog (https://www.ebi.ac.uk/gwas/downloads/summary‐statistics), SSC‐A on CD4^+^ corresponds to SSC‐A on CD4^+^ T cell. These findings suggest a preliminary conclusion that ZDHHC5 may promote the development of GAD through mediation by SSC‐A on CD4^+^ T cell (*β*_12_p = 36.4%).

**TABLE 3:** Effects of ZDHHC5 on GAD mediated by immune cell traits. (***β*_all_**: the total effect of ZDHHC5 on GAD, ***β*_1_**: the direct effect of ZDHHC5 on immune cell traits, ***β*_2_**: the independent effect of immune cell traits on GAD, and ***β*_12_**: mediating effect, calculated as ***β*_12_** = ***β*_1_** × ***β*_2_**. ***β*_12p_**: The proportion of the mediating effect on the total effect).

Trait	*β*_all_	*β*_1_	*β*_2_	*β*_12_	*β*_12p_
**CD28 + CD45RA − CD8br %CD8br**	0.096	0.220	0.043	0.009	0.098
**CD25 on CD39 + CD4+**	0.096	0.156	0.030	0.005	0.048
**CD4 on CD4 +**	0.096	0.263	− 0.108	− 0.028	− 0.294
**CD4 on CM CD4 +**	0.096	0.322	− 0.106	− 0.034	− 0.356
**CD4 on naive CD4 +**	0.096	0.135	− 0.106	− 0.014	− 0.149
**CD4 on CD45RA + CD4+**	0.096	0.261	− 0.100	− 0.026	− 0.271
**SSC‐A on CD4+**	0.096	0.286	0.123	0.035	0.364

Abbreviations: *β*all = the total effect of ZDHHC5 on GAD; *β*1 = the direct effect of ZDHHC5 on immune cell traits; *β*2: the independent effect of immune cell traits on GAD; *β*12: mediating effect, calculated as *β*12 = *β*1 × *β*2; *β*12p: the proportion of the mediating effect on the total effect.

**TABLE 4:** Effects of ZDHHC13 on GAD mediated by immune cell traits.

Trait	*β*_all_	*β*_1_	*β*_2_	*β*_12_	*β*_12p_
**EM CD4 + %T cell**	0.161	0.197	0.086	0.017	0.105
**CD20 on B cell**	0.161	0.162	− 0.078	− 0.013	− 0.079
**CD27 on CD24 + CD27 +**	0.161	0.190	0.046	0.009	0.054
**CD27 on IgD + CD24 +**	0.161	0.257	0.060	0.015	0.096
**CD27 on IgD + CD38 − unsw mem**	0.161	0.341	0.057	0.020	0.122
**CD27 on IgD − CD38 −**	0.161	0.166	0.068	0.011	0.070
**CD27 on IgD − CD38 dim**	0.161	0.235	0.049	0.011	0.071
**CD27 on memory B cell**	0.161	0.196	0.065	0.013	0.079
**CD27 on unsw mem**	0.161	0.256	0.062	0.016	0.098
**CD27 on sw mem**	0.161	0.219	0.048	0.011	0.066
**HLA DR on plasmacytoid DC**	0.161	0.172	0.021	0.004	0.022
**HLA DR on DC**	0.161	0.240	0.022	0.005	0.033

## 4 Discussion

In this study, large‐scale genetic datasets and MR methodologies were employed to investigate the causal relationship between 22 palmitoylation‐eQTL genes and GAD, thereby elucidating molecular pathways potentially mediated by immune cells. Notably, ZDHHC5 was identified as a genetic risk factor for GAD via upregulation of SSC‐A on CD4+ T cells (OR = 1.101, *p* = 0.017, *β*_12_p = 36.4%). The robustness of the association between palmitoylation‐eQTL genes and GAD was substantiated through sensitivity analyses, which revealed no evidence of horizontal pleiotropy or outlier bias. Moreover, Cochran's Q test in the MR framework and the HEIDI test in the SMR analysis both indicated the absence of significant heterogeneity between ZDHHC5 and GAD.

Palmitoylation is a vital post‐translational protein modification that dynamically regulates the protein lifecycle and function by facilitating membrane interactions and transport, as well as modulating protein‐protein interactions and enzymatic activity (Bijlmakers and Marsh 2003, Cho and Park 2016, Ko and Dixon 2018, Smotrys and Linder 2004). The palmitoylation process is governed by palmitoylation enzymes (e.g., ZDHHC5, ZDHHC13) and depalmitoylation enzymes (e.g., PPT1, PPT2), and is characterized by its reversibility (Chen et al. 2024). In eukaryotic cells, S‐palmitoylation reactions are catalyzed by protein acyltransferases (PATs), which generally possess a conserved aspartate–histidine–histidine–cysteine (DHHC) tetrapeptide motif and zinc finger domains, thereby classifying them as members of the ZDHHC family (Lemonidis et al. 2015). Each PAT exhibits a distinct subcellular localization, with the majority concentrated in the Golgi apparatus (Ohno et al. 2006), while others are distributed across the endoplasmic reticulum and plasma membrane (Woodley and Collins 2021).

Palmitoylation, recognized as a pivotal post‐translational modification, exerts multifaceted regulatory effects within both innate and adaptive immune systems by dynamically modulating the subcellular localization and functional activity of critical immune cell proteins. In macrophages, S‐palmitoylation facilitates the endoplasmic reticulum processing, secretory trafficking, and lipid raft targeting of CD36 (Thorne et al. 2010), thereby orchestrating macrophage‐mediated phagocytosis of pathogenic ligands such as oxLDL, amyloid‐β, and myelin, which are implicated in pathological processes including atherosclerosis, Alzheimer's disease, and multiple sclerosis (Penberthy and Ravichandran 2016, Grajchen et al. 2020). Empirical evidence has indicated that inflammation‐associated hyaluronan is cleared by macrophages in a CD44‐dependent manner, wherein the palmitoylation of CD44 is indispensable for hyaluronan internalization (Lee‐Sayer et al. 2015, Thankamony and Knudson 2006). Moreover, palmitoylation is essential for the membrane localization of nucleotide‐binding oligomerization domain proteins—intracellular receptors responsible for detecting cytoplasmic bacterial peptidoglycans—and for the subsequent activation of NF‐κB and MAPK signaling cascades in macrophages (Lu et al. 2019, Moreira and Zamboni 2012). In B cells, palmitoylation of CD81 augments the strength and persistence of antigen receptor signaling by stabilizing the positioning of the T cell receptor (BCR)/CD19/CD21 complex within lipid rafts (Cherukuri et al. 2004), while palmitoylation of the HGAL protein enhances its interaction with Syk kinase to promote BCR signaling, concurrently alleviating its suppression of chemokine‐induced cell motility (Lu et al. 2015). This reversible modification underscores how palmitoylation precisely coordinates B cell activation and migration via spatially and temporally specific modulation of protein subcellular localization. In T cells, palmitoylation critically influences activation and functionality by regulating the localization and interaction of T cell receptor (TCR) signaling components. It has been demonstrated that the dynamic palmitoylation of ZAP‐70 is temporally synchronized with TCR signal transduction, a prerequisite for its substrate affinity and downstream signaling, thereby directly shaping the initiation and amplitude of T cell activation (Tewari et al. 2021). The palmitoylation of CD4/CD8 co‐receptors enhances TCR sensitivity by promoting membrane localization and facilitating synergistic interactions with the MHC‐TCR complex (Crise and Rose 1992, Fragoso et al. 2003, Arcaro et al. 2000). Additionally, ZDHHC21‐catalyzed acylation is vital for the differentiation of peripheral CD4+ T cells into Th1/Th2/Th17 effector lineages, elucidating the pivotal regulatory role of this modification in T cell‐mediated adaptive immune responses (Bieerkehazhi et al. 2022). Collectively, these findings delineate the integral functions of palmitoylation in immune cell activation, migration, and differentiation, offering a conceptual framework for targeting lipid modifications in the modulation of immune‐related disorders.

Increasing evidence suggests the involvement of immune cell‐mediated pathogenic mechanisms across a spectrum of psychiatric disorders (Özyurt and Binici 2019, Réus et al. 2015, Brown et al. 2009, Benros et al. 2013). Numerous investigations have established that increased concentrations of inflammatory signals, encompassing IL‐1, IL‐2, IL‐6, IFN‐γ, and TNF‐α, are positively associated with GAD symptoms (Tang et al. 2018, Michopoulos et al. 2017, Yang et al. 2017, Hou et al. 2017), and that immunomodulatory interventions targeting specific cytokines may alleviate these symptoms (Uguz et al. 2009). Moreover, elevated cytokine concentrations have been correlated with resistance to antidepressant treatments and unfavorable clinical outcomes in psychiatric populations (Haroon et al. 2018, Chamberlain et al. 2019, Kose et al. 2021). Nevertheless, certain studies have reported no significant differences in inflammatory mediators like IL‐6 and TNF‐α between individuals with anxiety disorders and healthy controls (Vogelzangs et al. 2013), thereby highlighting the necessity for further elucidation of the underlying pathophysiological mechanisms. A substantial body of clinical and translational research has indicated that exposure to exogenous cytokines or inflammatory stimuli may initiate or intensify depressive and anxiety‐like behaviors by disrupting neurotransmitter systems and neural circuit integrity, thus implicating these processes in the etiology of various neuropsychiatric disorders (Felger and Lotrich 2013, Dantzer et al. 2008, Miller and Raison 2016). In addition, elevated cytokine levels, aberrant activation of signaling pathways, and infiltration of peripheral immune cells into the brain parenchyma have been identified in patients with psychiatric conditions (Torres‐Platas et al. 2014, North et al. 2021, Cai et al. 2020, Zhu et al. 2022). As the primary immune effector cells of the central nervous system (CNS), microglia play a pivotal role in this process: psychosocial stress can directly induce their activation into an M1‐like pro‐inflammatory phenotype (Réus et al. 2015, Miller and Raison 2016), releasing chemokines such as C‐C motif chemokine ligand 2 (CCL2) to recruit activated peripheral myeloid cells into the brain parenchyma via cellular mechanisms (Cai et al. 2020). These infiltrating macrophages and activated microglia engage in a positive feedback loop, collectively amplifying the central inflammatory cascade even without overt blood‐brain barrier disruption (Cai et al. 2020). Specifically, peripheral inflammatory challenges have been shown to activate brain regions encompassing the amygdala, anterior cingulate cortex, and insula, thereby enhancing neuronal excitability and inducing local synthesis of pro‐inflammatory cytokines (IL‐1β, IL‐6, TNF‐α), while microglia‐mediated dysregulation of the tryptophan‐kynurenine metabolic pathway further perturbs monoaminergic neurotransmission (e.g., serotonin) and glutamatergic homeostasis, ultimately provoking anxiety‐related behaviors (Chamberlain et al. 2019, Felger 2018, Munshi and Rosenkranz 2018, Engler et al. 2011). These findings imply that immune cytokines substantially contribute to the pathogenesis of anxiety and other psychiatric disorders by disrupting neurotransmitter dynamics and neural circuitry through inflammatory signaling pathways.

As a contributing factor to GAD, ZDHHC5 has been shown to upregulate SSC‐A on CD4+ T cells, thereby facilitating disease onset. This finding aligns with previous evidence indicating that palmitoylation‐mediated regulation of immune cell activation, subsequently via inflammatory stimulation, contributes to the development of psychiatric disorders. However, the precise mechanism by which ZDHHC5 contributes to GAD pathogenesis remains unclear. ZDHHC5 localizes to the cell membrane and modulates key proteins involved in T cell signaling and activation (Woodley and Collins 2021, Tewari et al. 2021). Side scatter (SSC) values principally reflect the complexity and granularity of intracellular structures; cells exhibiting greater size and internal complexity tend to demonstrate elevated SSC readings. The combination of forward scatter area (FSC‐A) and SSC‐A enables the sorting of CD4+ T cells, allowing for the exclusion of cellular debris and nonviable cells (Chen et al. 2024). As central effectors of the adaptive immune response, CD4+ T lymphocytes exhibit dual immunological roles by modulating inflammatory cascades through the secretion of both pro‐inflammatory cytokines (e.g., IL‐2, IFN‐γ, IL‐5, IL‐17, and TNF‐α) and anti‐inflammatory cytokines (e.g., IL‐4, IL‐10) (Cenerenti et al. 2022). Under inflammatory pathological conditions, CD4+ T cells can be induced to infiltrate the brain parenchyma by traversing the microvasculature of the blood‐brain barrier, resulting in disruption of astrocytic end‐feet and the basement membrane (Wang et al. 2022). Prior studies have reported that patients with anxiety disorders exhibit elevated lymphocyte and T cell counts, along with heightened T cell sensitivity (Boscarino and Chang 1999). Additionally, the depletion of CD4+ T cells has been found to confer resistance to stress‐induced anxiety‐like behaviors in murine models (Fan et al. 2019). These data further support ZDHHC5 upregulation of SSC‐A on CD4+ T cells as contributing to GAD pathogenesis. Stress exposure has also been shown to induce mitochondrial fission and metabolic disturbances in peripheral CD4+ T cells, thereby promoting excessive production of the purine metabolite xanthine. This metabolite, acting through adenosine A1 receptors located on oligodendrocytes within the left amygdala, has been linked to the manifestation of anxiety‐like behaviors (Fan et al. 2019). Drawing upon current and prior findings, ZDHHC5 is hypothesized to regulate protein palmitoylation within CD4+ T cells, potentially enhancing their activation and stimulating the secretion of IL‐2, IFN‐γ, IL‐5, IL‐17, and TNF‐α. Furthermore, ZDHHC5 may contribute to CD4+ T cell mitochondrial fission and xanthine overproduction. Elevated levels of pro‐inflammatory cytokines and xanthine acting upon the amygdala and associated brain regions may collectively provoke anxiety.

This study confers distinct methodological advantages. While traditional GWAS identifies non‐causal associations and candidate gene studies rely on prespecified biological hypotheses, MR leverages genetic instruments to minimize confounding/reverse causation. Critically, by integrating eQTL data with SMR validation, we pinpointed ZDHHC5 and ZDHHC13 as bona fide risk genes, surpassing the limited resolution of standalone GWAS. Through two‐step MR mediation analysis, we further quantified SSC‐A on CD4⁺ T cells as a mechanistic mediator specifically linking ZDHHC5 to GAD (mediation proportion: 36.4%). This integrative approach—synthesizing causal gene prioritization with immune pathway dissection—delivers novel mechanistic insights into GAD etiology, complementing limitations of prior methods.

This study has several potential limitations. First, to minimize population stratification bias and satisfy key MR design assumptions, the GWAS and eQTL data utilized were primarily derived from individuals of European ancestry. Consequently, the genetic architecture of GAD, the regulatory patterns of ZDHHC5, and their association with CD4⁺ T cells may differ in other ancestral populations. The generalizability of our findings to non‐European populations requires validation in diverse cohorts. Second, although pleiotropy was rigorously addressed through sensitivity analyses (MR‐Egger regression, MR‐PRESSO), residual confounding via unidentified pathways cannot be entirely excluded. We mitigated such bias by employing stringent IV selection criteria, Steiger directionality testing, validation across multiple MR methods (IVW, weighted median, etc.), and the HEIDI test. Third, the eQTL data employed originated from whole blood. While blood is a key relevant tissue for immune cells, the expression and regulation of palmitoylation‐related genes, such as ZDHHC5, within the CNS may differ. Our results likely reflect peripheral immune‐mediated mechanisms but may overlook CNS‐specific effects. Future investigations incorporating brain‐specific eQTL data are crucial to explore CNS‐specific influences. Furthermore, SSC‐A reflects cellular granularity/complexity and serves as an indirect indicator of CD4⁺ T cell activation status, rather than a direct measurement. Critically, while MR provides stronger evidence for causality compared to observational studies, it estimates the effect of genetic liability to the exposure, not necessarily the direct physiological effect itself. Therefore, further experimental validation is required to substantiate the regulatory role of ZDHHC5 in CD4⁺ T cell function and GAD.

## 5 Conclusion

In this study, MR methods were employed to elucidate a causal association between protein palmitoylation and GAD, with immune cells identified as key mediating components. Genetic evidence indicated that ZDHHC5 functions as a risk gene for GAD, with the effects mediated through SSC‐A on CD4⁺ T cells. These observations suggest a novel “palmitoylation‐immune‐neural cascade” framework, redirecting the mechanistic focus of anxiety pathogenesis from traditional monoaminergic or hypothalamic‐pituitary‐adrenal axis models toward alternative signaling pathways. This conceptual advancement has yielded potential therapeutic targets for GAD, particularly strategies aimed at modulating palmitoylation pathways and immune function.

## Author Contributions

**Fuqiang Sun**: conceptualisation, writing – original draft. **Gengchen Lu**: methodology, formal analysis, software. **Nannan Li**: conceptualization, writing – original draft. **Kun Dai**: methodology, data curation. **Zhe Ren**: data curation, resources. **Ruimin Ma**: data curation, resources. **Yifei Feng**: validation. **Shuo Zong**: validation. **Bin Cheng**: writing – review and editing.

## Ethics Statement

This study did not require ethical review and approval according to local laws and institutional requirements. Written informed consent from participants or their legal guardians/next of kin was not required for participation in this study in accordance with national legislation and institutional guidelines.

## Peer Review

The peer review history for this article is available at https://publons.com/publon/10.1002/brb3.70818

## Supporting information




**Supplementary Figures**: brb370818‐sup‐0001‐Figures.docx

**Supplementary Tables**: brb370818‐sup‐0002‐Tables.xls

## Data Availability

The datasets utilised in this study are accessible from online repositories. Repository names and accession numbers are provided in the article.
