# Supplementary material for: ZDHHC5 Mediates Immune Dysregulation Driving Generalized Anxiety Disorder Risk
Source: Brain Behav. 2025 Sep 2;15(9):e70818. doi: 10.1002/brb3.70818 (PMC12405600; doi:10.1002/brb3.70818)
Supplement: Supplementary file 1 — Supplementary Figures: brb370818‐sup‐0001‐Figures.docx [file BRB3-15-e70818-s001.docx]

a b


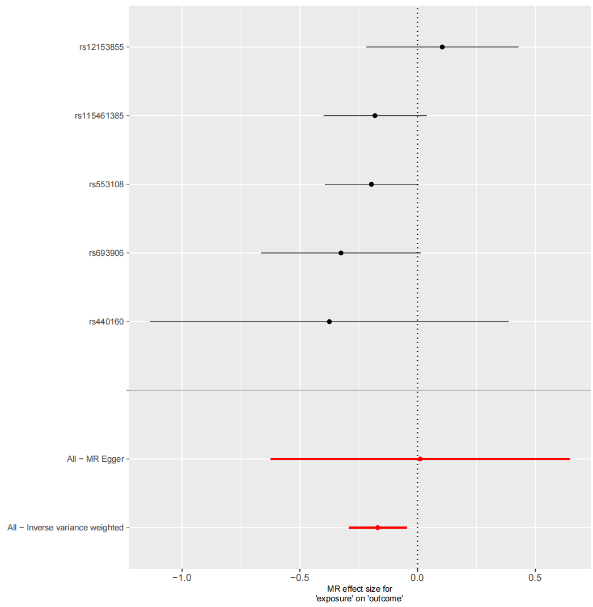

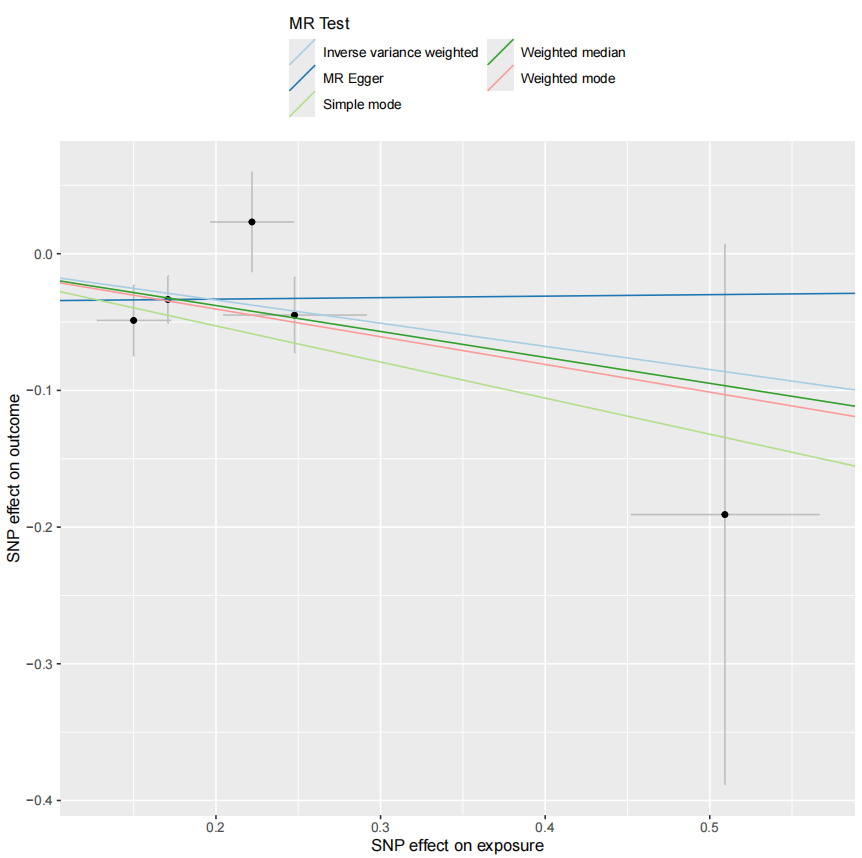


c d


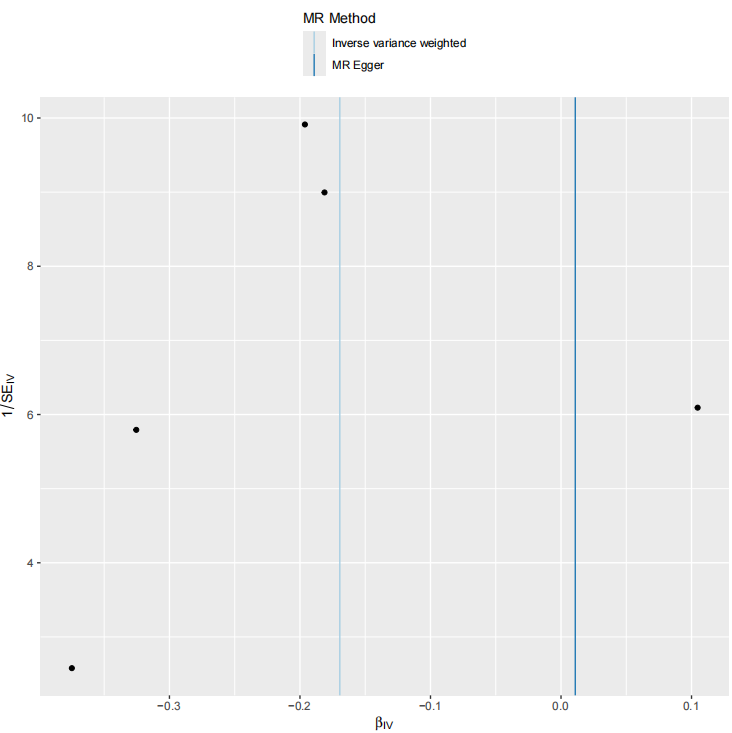

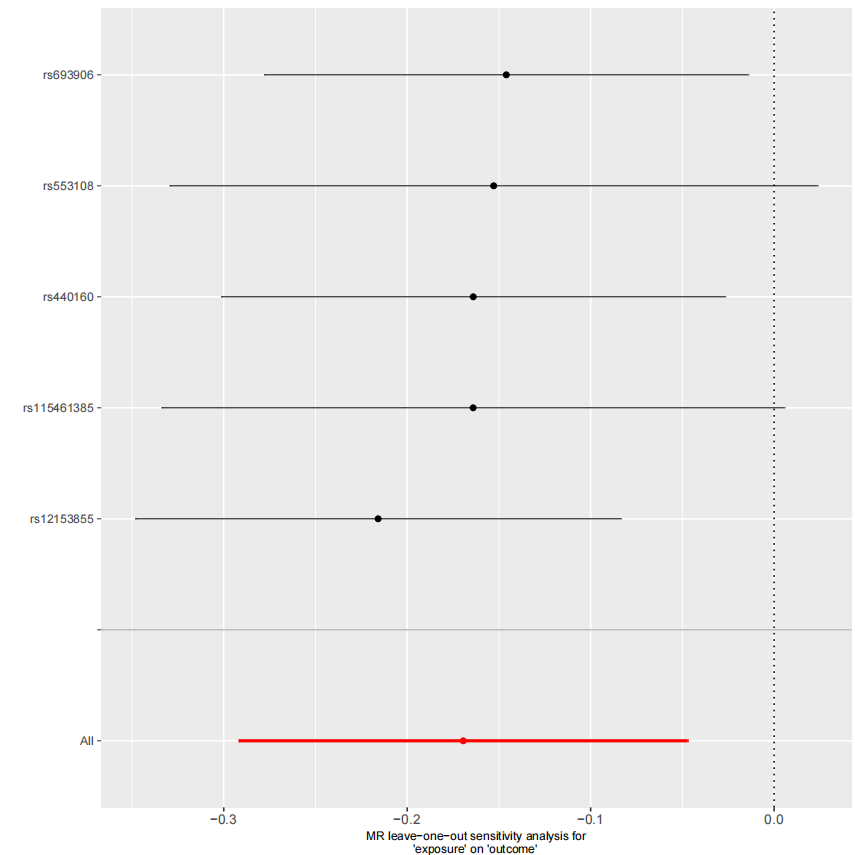


Supplementary Figure S1 Forest plot (a), scatter plot(b), funnel plot (c) and leave-one-out analysis (d) of SNPs associated with PPT2 on GAD.

a b


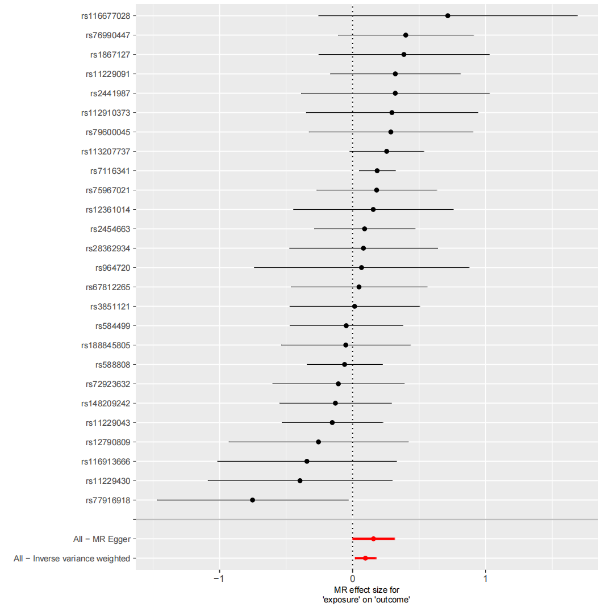

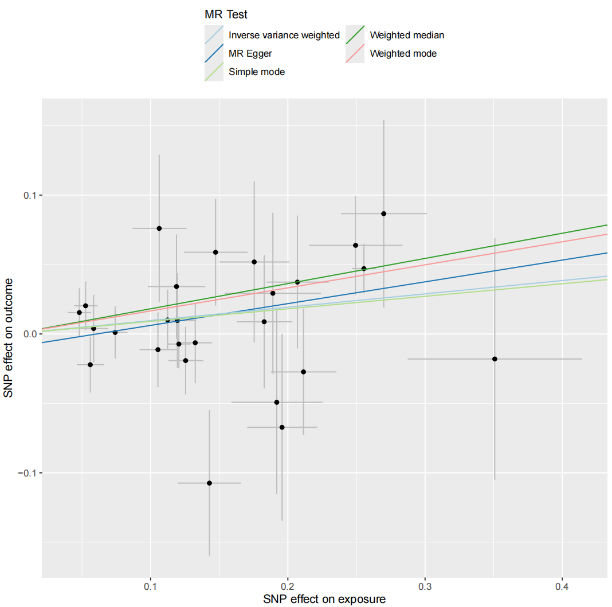


c d


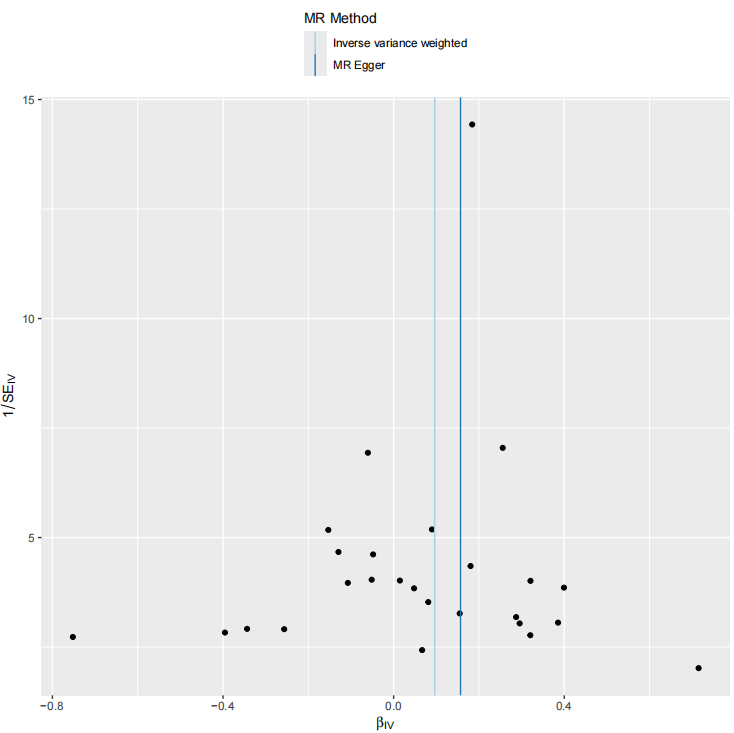

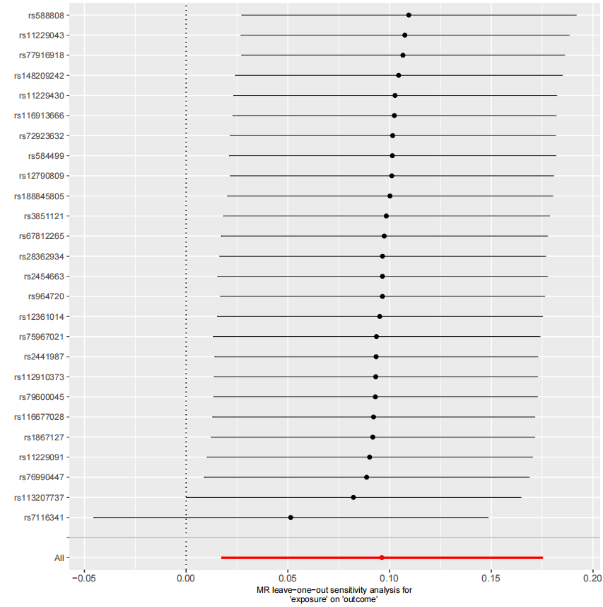


Supplementary Figure S2 Forest plot (a), scatter plot(b), funnel plot (c) and leave-one-out analysis (d) of SNPs associated with ZDHHC5 on GAD.

a b


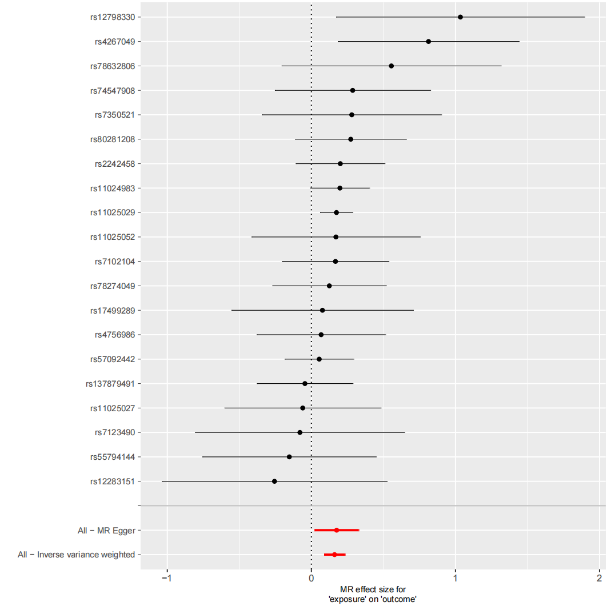

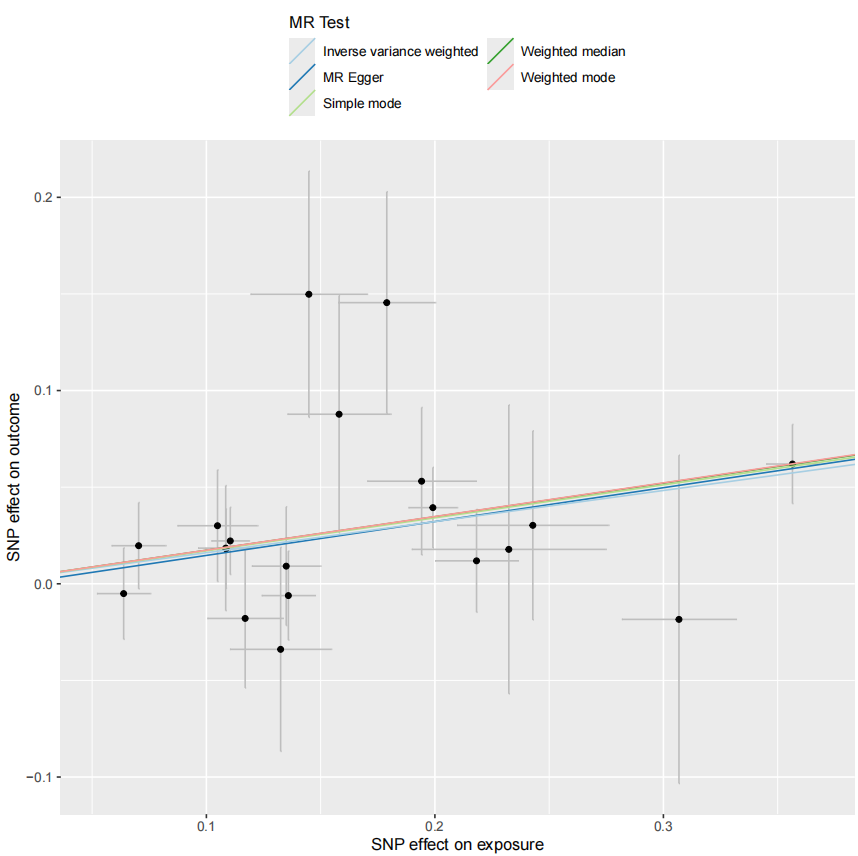


c d


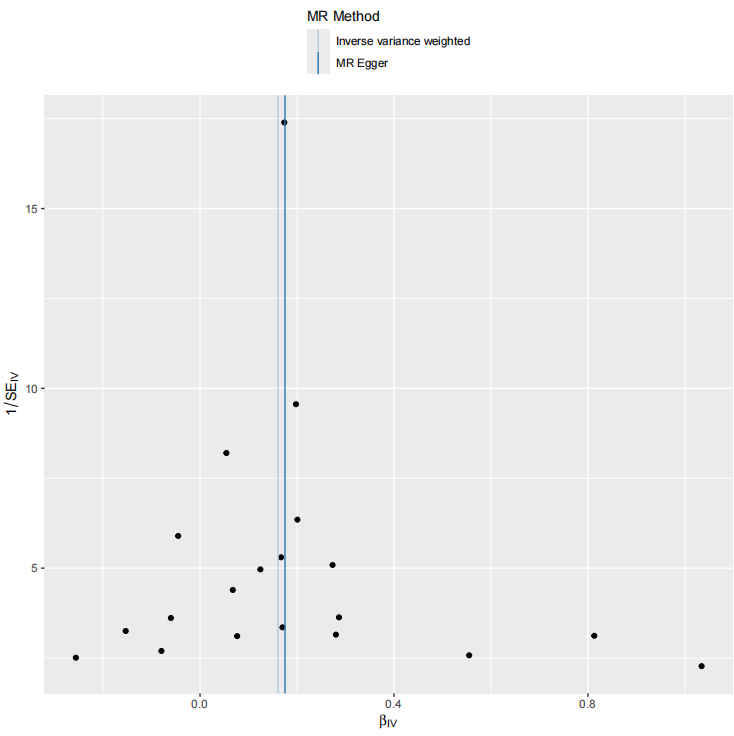

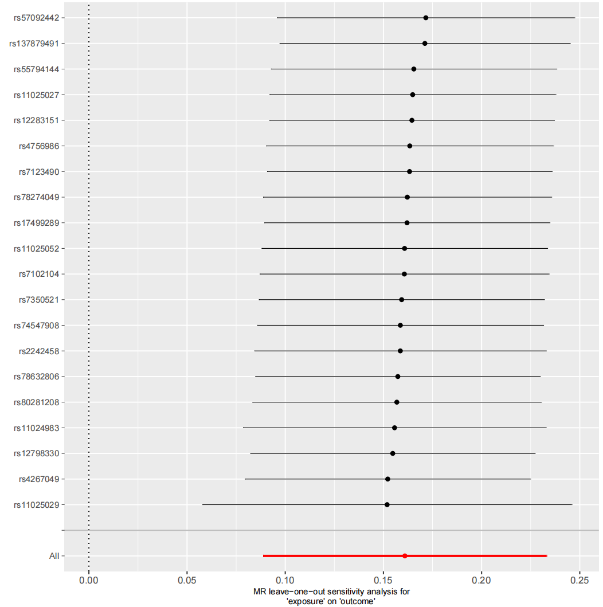


Supplementary Figure S3 Forest plot (a), scatter plot(b), funnel plot (c) and leave-one-out analysis (d) of SNPs associated with ZDHHC13 on GAD.

a b


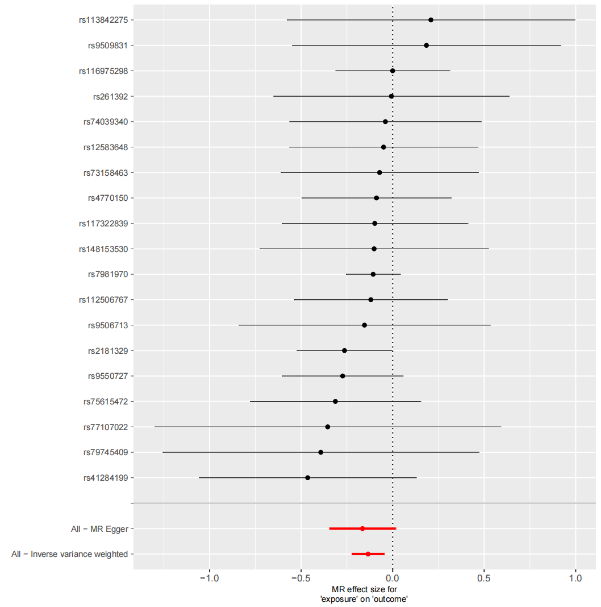

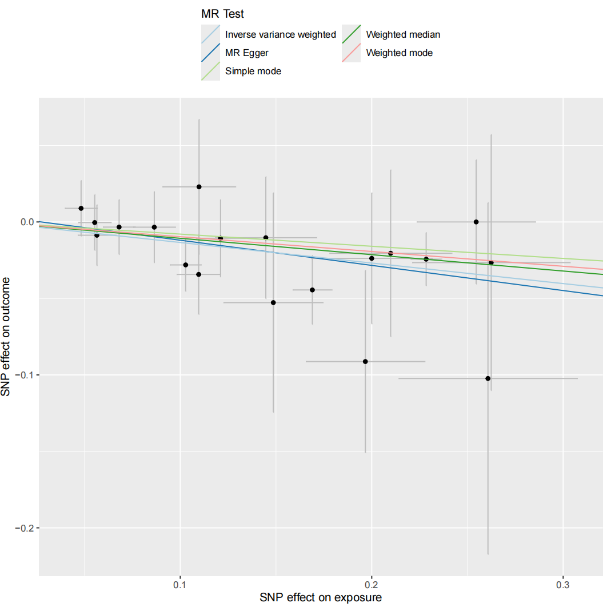


c d


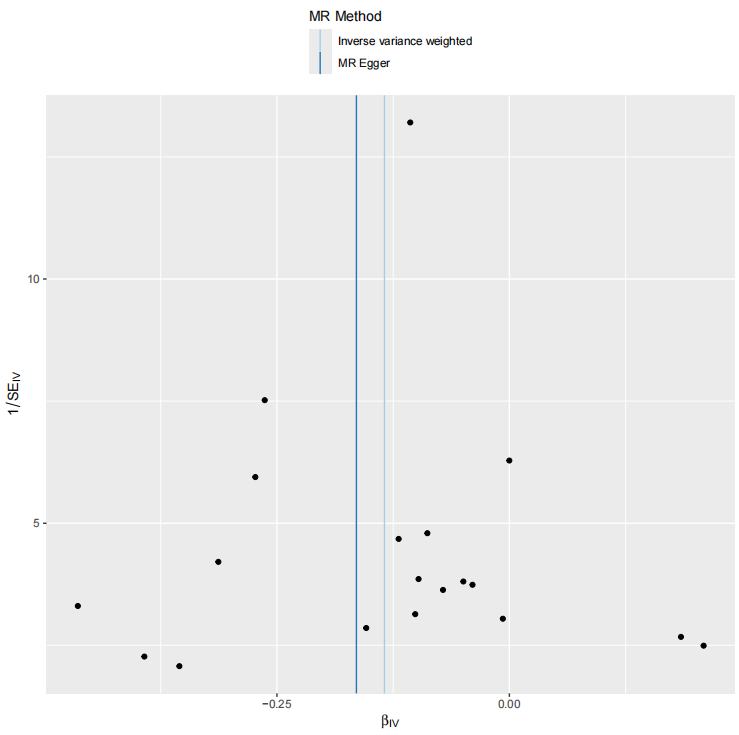

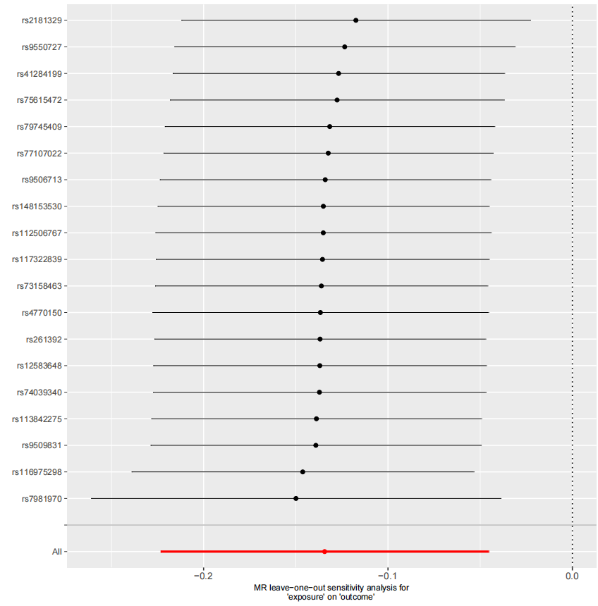


Supplementary Figure S4 Forest plot (a), scatter plot(b), funnel plot (c) and leave-one-out analysis (d) of SNPs associated with ZDHHC20 on GAD.

a b


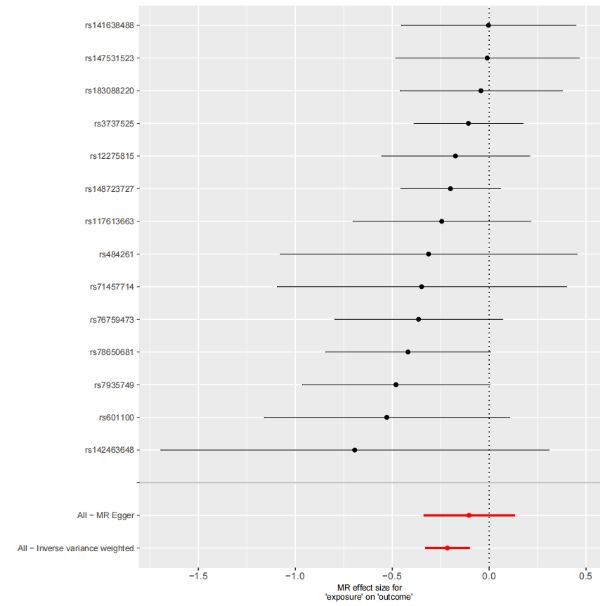

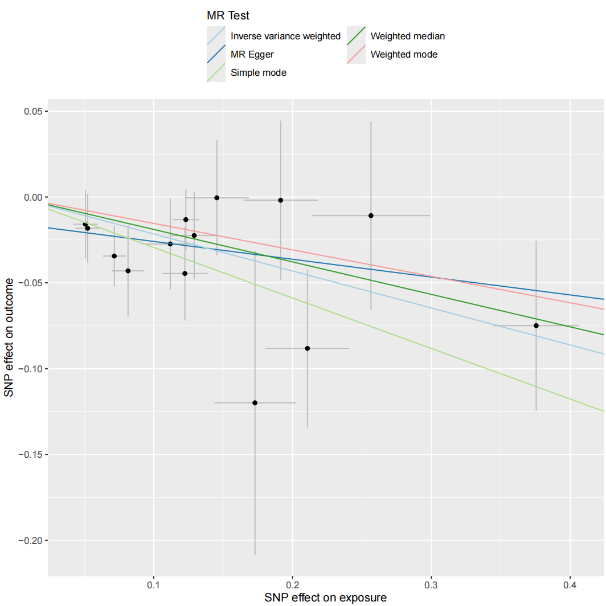


c d


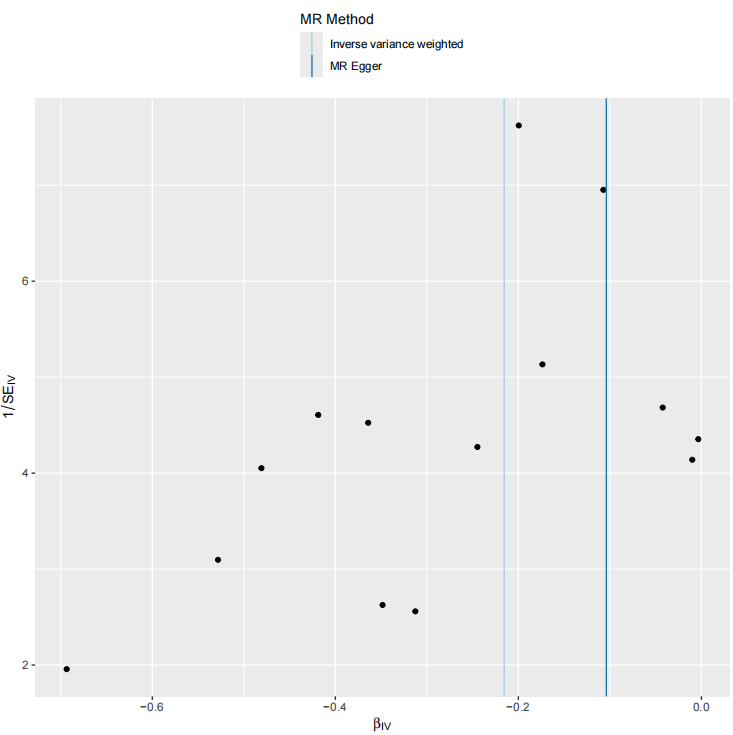

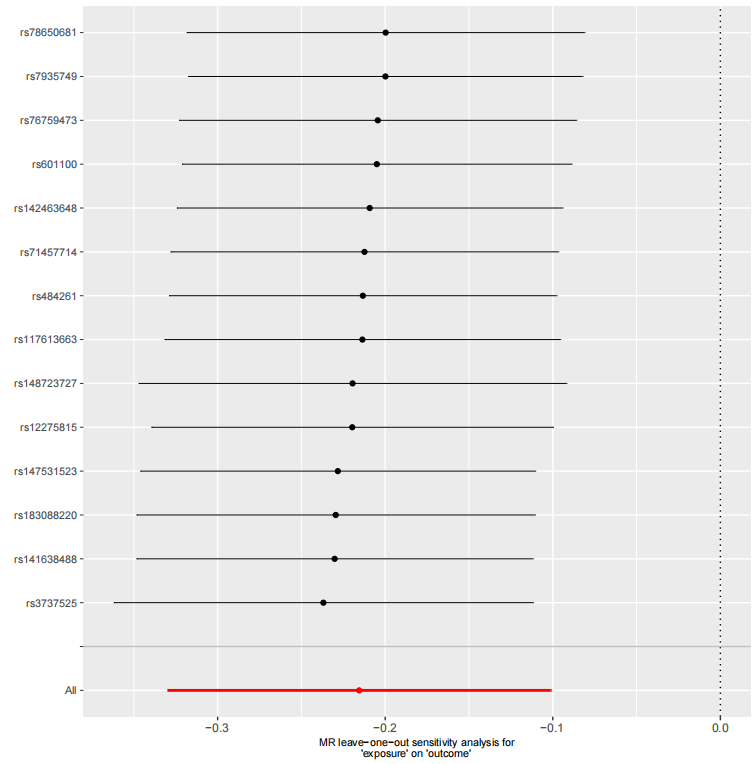


Supplementary Figure S5 Forest plot (a), scatter plot(b), funnel plot (c) and leave-one-out analysis (d) of SNPs associated with ZDHHC24 on GAD.
